# Risk factors associated with newly diagnosed attention-deficit/hyperactivity disorder in adults: a retrospective case-control study

**DOI:** 10.1186/s12888-023-05359-7

**Published:** 2023-11-23

**Authors:** Jeff Schein, Martin Cloutier, Marjolaine Gauthier-Loiselle, Rebecca Bungay, Emmanuelle Arpin, Annie Guerin, Ann Childress

**Affiliations:** 1 Otsuka Pharmaceutical Development & Commercialization, Inc, 508 Carnegie Center, Princeton, NJ 08540 USA; 2 Analysis Group, Inc, 1190 avenue des Canadiens-de-Montréal, Tour Deloitte, Suite 1500, Montréal, QC H3B 0G7 Canada; 3 Center for Psychiatry and Behavioral Medicine, 7351 Prairie Falcon Rd STE 160, Las Vegas, NV 89128 USA

**Keywords:** Attention-deficit/hyperactivity disorder, Anxiety disorder, Depressive disorder, Comorbidity, Risk factor

## Abstract

**Background:**

Knowledge of risk factors for attention-deficit/hyperactivity disorder (ADHD) may facilitate early diagnosis; however, studies examining a broad range of potential risk factors for ADHD in adults are limited. This study aimed to identify risk factors associated with newly diagnosed ADHD among adults in the United States (US).

**Methods:**

Eligible adults from the IQVIA PharMetrics® Plus database (10/01/2015-09/30/2021) were classified into the ADHD cohort if they had ≥ 2 ADHD diagnoses (index date: first ADHD diagnosis) and into the non-ADHD cohort if they had no observed ADHD diagnosis (index date: random date) with a 1:3 case-to-control ratio. Risk factors for newly diagnosed ADHD were assessed during the 12-month baseline period; logistic regression with stepwise variable selection was used to assess statistically significant association. The combined impact of selected risk factors was explored using common patient profiles.

**Results:**

A total of 337,034 patients were included in the ADHD cohort (mean age 35.2 years; 54.5% female) and 1,011,102 in the non-ADHD cohort (mean age 44.0 years; 52.4% female). During the baseline period, the most frequent mental health comorbidities in the ADHD and non-ADHD cohorts were anxiety disorders (34.4% and 11.1%) and depressive disorders (27.9% and 7.8%). Accordingly, a higher proportion of patients in the ADHD cohort received antianxiety agents (20.6% and 8.3%) and antidepressants (40.9% and 15.8%). Key risk factors associated with a significantly increased probability of ADHD included the number of mental health comorbidities (odds ratio [OR] for 1 comorbidity: 1.41; ≥2 comorbidities: 1.45), along with certain mental health comorbidities (e.g., feeding and eating disorders [OR: 1.88], bipolar disorders [OR: 1.50], depressive disorders [OR: 1.37], trauma- and stressor-related disorders [OR: 1.27], anxiety disorders [OR: 1.24]), use of antidepressants (OR: 1.87) and antianxiety agents (OR: 1.40), and having ≥ 1 psychotherapy visit (OR: 1.70), ≥ 1 specialist visit (OR: 1.30), and ≥ 10 outpatient visits (OR: 1.51) (all p < 0.05). The predicted risk of ADHD for patients with treated anxiety and depressive disorders was 81.9%.

**Conclusions:**

Mental health comorbidities and related treatments are significantly associated with newly diagnosed ADHD in US adults. Screening for patients with risk factors for ADHD may allow early diagnosis and appropriate management.

## Background

Attention-deficit/hyperactivity disorder (ADHD) is a debilitating neurodevelopmental condition with an estimated prevalence of 4.4% among adults in the United States (US) [1]. ADHD is traditionally perceived as a childhood disorder [2]; hence, underdiagnosis, delayed diagnosis, and undertreatment of ADHD are believed to be common among adults [3, 4].

The diagnostic challenges of ADHD are partially attributable to the frequent comorbid mental disorders [5, 6]. Certain mental health comorbidities, such as anxiety and depressive disorders, share overlapping symptoms with ADHD [7, 8], potentially leading to misdiagnosis or delayed diagnosis. Studies have suggested that about one-fifth of adults seeking psychiatric services and reporting for other mental health conditions were later found to have ADHD [9–11]. The World Health Organization Mental Health Survey has also reported that among US adults with ADHD identified through diagnostic interviews, approximately half had received some form of treatment for their emotional or behavioral problems in the past year, but only 13.2% were treated specifically for ADHD [12]. Clinicians’ lack of awareness or training on adult ADHD may also hinder ADHD diagnosis [4]. A US medical record-based study found that 56% of adults with ADHD had not received a prior diagnosis of the condition despite complaining about ADHD symptoms to other healthcare professionals in the past [13]. Other reasons adding to the diagnostic challenge of ADHD in adults may include patient’s fear of stigma and masking behaviors developed over the years [4, 14].

ADHD is associated with a wide range of psychosocial, functional, and occupational problems in adults [15]. A delay in diagnosis, or undiagnosed and ultimately untreated ADHD, may lead to poor clinical and functional outcomes even if comorbidities are treated [16]. Conversely, early identification of ADHD may allow better symptom management and improve patient functioning and quality of life. To facilitate diagnosis, risk factors are commonly used to predict disease development and aid clinicians to identify at-risk patients [17]. However, there is a paucity of large studies examining a broad range of potential risk factors for an ADHD diagnosis in adults. Prior studies have reported certain patient characteristics, such as presence of anxiety disorders, depressive disorders, sleep impairments, eating disorders, and childhood illnesses or health events (e.g., obesity, head injuries, infections) that may be associated with ADHD [18–23]. Yet, most of these studies have examined a single or a few factors, and many were conducted in pediatric ADHD populations primarily outside of the US.

Knowledge on patient characteristics associated with a higher risk of ADHD in adults and the patient journey prior to a clinical ADHD diagnosis may facilitate early diagnosis and the provision of appropriate management. The current study was conducted to identify risk factors for newly diagnosed ADHD in adult patients using a large claims database in the US. The potential utility of the results was also demonstrated through exploring the combined impact of selected risk factors on ADHD risk prediction using fictitious common patient profiles.

## Methods

### Data source

Data from the IQVIA PharMetrics® Plus (IQVIA) database covering the period of October 1, 2015, to September 30, 2021, were used. The IQVIA database contains integrated claims data of over 190 million beneficiaries across the US and includes information on inpatient and outpatient diagnoses and procedures, prescription fills, patients’ pharmacy and medical benefits, inpatient stays, and provider details. Additional data elements encompass dates of service, demographic variables, plan type, payer type, and start and stop dates of health plan enrollment. Data are de-identified and comply with the patient requirements of the Health Insurance Portability and Accountability Act (HIPAA); therefore, no review by an institutional review board nor informed consent was required per Title 45 of CFR, Part 46.101(b)(4) [24].

### Study design and patient populations

A retrospective case-control study design was used. Eligible adults were classified into two cohorts based on the presence of ADHD diagnoses (International Classification of Diseases, Tenth Revision, Clinical Modification [ICD-10-CM] F90.x): the *ADHD cohort* comprised patients with ≥ 2 ADHD diagnoses recorded on a medical claim on distinct dates at any time during their continuous health plan enrollment; and the *non-ADHD cohort* comprised patients without any ADHD diagnoses recorded on a medical claim at any time during their continuous health plan enrollment. To account for large differences in sample size and to retain statistical power, a 1:3 case-to-control ratio was used. Specifically, eligible patients were randomly selected into the non-ADHD cohort such that the total number of patients in the non-ADHD cohort was three times that of the ADHD cohort.

The index date was defined as the first observed ADHD diagnosis among the ADHD cohort and a randomly selected date among the non-ADHD cohort. To allow sufficient time to capture potential risk factors for ADHD, patients were required to have ≥ 12 months of continuous health plan enrollment prior to the index date. The baseline period was defined as the 12 months pre-index.

### Study measures and outcomes

Patient characteristics and potential risk factors for newly diagnosed ADHD were assessed during the baseline period for each cohort, separately. Potential risk factors considered in this study were identified through a targeted literature review and observable variables in the data and included demographic characteristics (i.e., age, sex, regions of residence, calendar year of index date), clinical characteristics (i.e., physical and mental health comorbidities), pharmacological treatments (i.e., medications for common ADHD comorbidities), healthcare resource utilization (i.e., number of psychotherapy, inpatient, emergency room, outpatient, and specialist [psychiatrist, neurologist] visits). Risk factors for ADHD in this study were identified from potential risk factors that had statistically significant association with newly diagnosed ADHD, as described in the next section.

### Statistical analyses

Descriptive statistics were used to summarize baseline patient characteristics and potential risk factors for newly diagnosed ADHD. Means, medians, and standard deviations (SDs) were reported for continuous variables; frequency counts and percentages were reported for categorical variables.

Univariate statistics were used to compare potential risk factors between the ADHD and non-ADHD cohorts. The magnitude of the difference between cohorts was assessed by calculating the standardized differences (std. diff.) for both continuous and categorical variables.

Logistic regression model with stepwise variable selection was used to assess statistically significant association between potential risk factors and ADHD diagnosis. Potential risk factors were eligible for inclusion in the logistic regression based on their univariate association with ADHD diagnosis (i.e., std. diff. >0.10). Potential risk factors presented in < 0.5% of the sample were discarded. Variables included in the last iteration of the stepwise selection process were considered as risk factors of the study outcome. The association between risk factors and ADHD diagnosis were reported as odds ratios (ORs) along with their 95% confidence intervals (CIs) and p-values.

To facilitate the interpretation of the regression analyses, the predicted risk of ADHD based on regression coefficient estimates was evaluated for six fictitious common patient profiles corresponding to patients who harbor selected combinations of ADHD risk factors. This exploratory analysis allowed for the estimation of how the risk of having ADHD would vary had the same person had additional risk factors but otherwise the same characteristics.

## Results

### Patient characteristics and potential risk factors

**Fig. 1:**
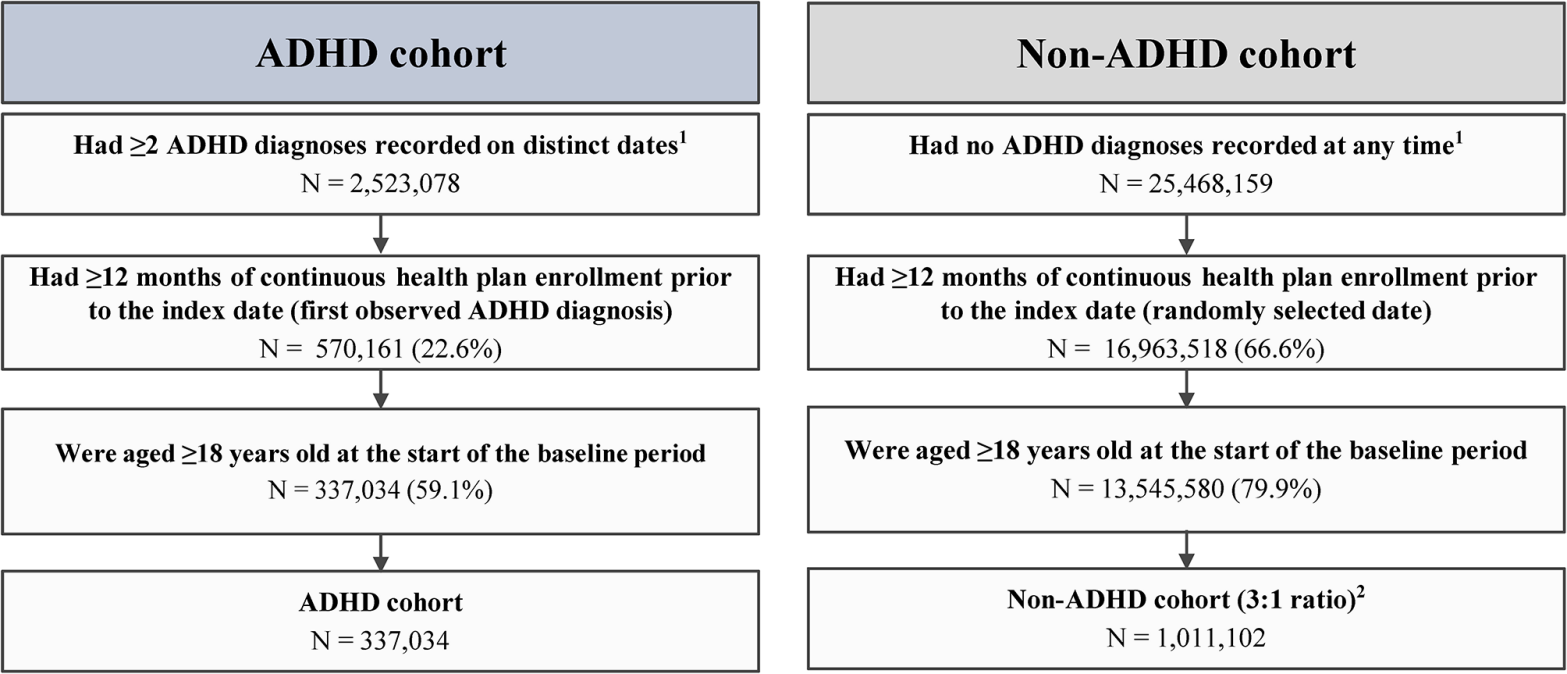
Sample selection flowchart. ADHD, attention-deficit/hyperactivity disorder; ICD-10-CM, International Classification of Diseases, Tenth Revision, Clinical Modification Notes: ^1^ADHD was defined as ICD-10-CM codes: F90.x ^2^Eligible patients were randomly selected into the non-ADHD cohort such that the total number of patients in the non-ADHD cohort is 3 times that of the ADHD cohort to account for large differences in sample size

The total sample comprised 1,348,136 patients, including 337,034 in the ADHD cohort and 1,011,102 in the non-ADHD cohort (Fig. 1). Table 1 presents the patient characteristics and potential risk factors (i.e., characteristics with a std. diff. >0.10) by cohort.

**Table 1:** Patient characteristics and potential risk factors

	ADHD cohort *N* = 337,034	Non-ADHD cohort *N* = 1,011,102	Std. diff. ^1^	
**Demographic characteristics as of index date**				
** Age (years), mean ± SD [median]**	**35.2 ± 11.8 [33.0]**	**44.0 ± 14.0 [44.4]**	**0.68** ^†^	
**Female,***n* (%)	**183,534 (54.5%)**	**529,559 (52.4%)**	**0.04**	
**Region,***n* (%)				
South	164,236 (48.7%)	429,656 (42.5%)	0.13^†^	
Midwest	78,231 (23.2%)	232,443 (23.0%)	0.01	
Northeast	51,532 (15.3%)	182,985 (18.1%)	0.08	
West	42,192 (12.5%)	157,260 (15.6%)	0.09	
Unknown	843 (0.3%)	8,758 (0.9%)	0.08	
** Calendar year of index date,***n* (%)				
2016	22,229 (6.6%)	132,614 (13.1%)	0.22^†^	
2017	75,965 (22.5%)	241,400 (23.9%)	0.03	
2018	65,717 (19.5%)	159,479 (15.8%)	0.10	
2019	63,377 (18.8%)	154,452 (15.3%)	0.09	
2020	62,201 (18.5%)	160,095 (15.8%)	0.07	
2021	47,545 (14.1%)	163,062 (16.1%)	0.06	
**Clinical characteristics** ^**2**^				
** Physical comorbidities**^**3**^, *n* (%)				
Hypertension	41,766 (12.4%)	215,470 (21.3%)	0.24^†^	
Obesity	33,664 (10.0%)	95,197 (9.4%)	0.02	
Chronic pulmonary disease	30,350 (9.0%)	73,149 (7.2%)	0.07	
Diabetes (mild)	10,799 (3.2%)	61,556 (6.1%)	0.14^†^	
** Mental health comorbidities**^**4**^, *n* (%)				
Anxiety disorders	115,827 (34.4%)	111,780 (11.1%)	0.58^†^	
Depressive disorders	94,137 (27.9%)	79,049 (7.8%)	0.54^†^	
Sleep-wake disorders	44,341 (13.2%)	77,595 (7.7%)	0.18^†^	
Trauma- and stressor-related disorders	41,690 (12.4%)	34,881 (3.4%)	0.34^†^	
Substance-related and addictive disorders	31,521 (9.4%)	50,711 (5.0%)	0.17^†^	
Bipolar and related disorders	21,565 (6.4%)	10,469 (1.0%)	0.29^†^	
Obsessive-compulsive and related disorders	5,239 (1.6%)	2,571 (0.3%)	0.14^†^	
Feeding and eating disorders	4,422 (1.3%)	1,843 (0.2%)	0.13^†^	
Personality disorders	3,073 (0.9%)	1,336 (0.1%)	0.11^†^	
** Number of mental health comorbidities**^**5**^, **mean ± SD [median]**	**1.2 ± 1.4 [1.0]**	**0.5 ± 0.9 [0.0]**	**0.65** ^**†**^	
0, *n* (%)	141,548 (42.0%)	716,043 (70.8%)	0.61^†^	
1, *n* (%)	79,901 (23.7%)	184,375 (18.2%)	0.14^†^	
≥ 2, *n* (%)	115,585 (34.3%)	110,684 (10.9%)	0.58^†^	
**Pharmacological treatments** ^**2**^				
Antidepressants, *n* (%)	137,753 (40.9%)	159,775 (15.8%)	0.58^†^	
Antianxiety agents, *n* (%)	69,375 (20.6%)	83,463 (8.3%)	0.36^†^	
Anticonvulsants, *n* (%)	54,375 (16.1%)	69,207 (6.8%)	0.29^†^	
Antipsychotics, *n* (%)	24,200 (7.2%)	15,620 (1.5%)	0.28^†^	
**Healthcare resource utilization** ^**2**^				
** Number of psychotherapy visits, mean ± SD [median]**	**2.9 ± 8.8 [0.0]**	**0.6 ± 4.0 [0.0]**	**0.34** ^**†**^	
0, *n* (%)	254,222 (75.4%)	956,723 (94.6%)	0.56^†^	
1–4, *n* (%)	33,272 (9.9%)	22,848 (2.3%)	0.32^†^	
5–9, *n* (%)	17,033 (5.1%)	11,494 (1.1%)	0.23^†^	
≥ 10, *n* (%)	32,507 (9.6%)	20,037 (2.0%)	0.33^†^	
** Number of inpatient admissions, mean ± SD [median]**	**0.1 ± 0.4 [0.0]**	**0.1 ± 0.3 [0.0]**	**0.04**	
0, *n* (%)	318,139 (94.4%)	963,301 (95.3%)	0.04	
1–4, *n* (%)	18,598 (5.5%)	47,326 (4.7%)	0.04	
5–9, *n* (%)	260 (0.1%)	452 (0.0%)	0.01	
≥ 10, *n* (%)	37 (0.0%)	23 (0.0%)	0.01	
** Number of days with emergency room visits, mean ± SD [median]**	**0.6 ± 1.7 [0.0]**	**0.4 ± 1.2 [0.0]**	**0.14** ^†^	
0, *n* (%)	226,524 (67.2%)	756,599 (74.8%)	0.17^†^	
1–4, *n* (%)	103,442 (30.7%)	243,834 (24.1%)	0.15^†^	
5–9, *n* (%)	5,902 (1.8%)	9,013 (0.9%)	0.08	
≥ 10, *n* (%)	1,166 (0.3%)	1,656 (0.2%)	0.04	
** Number of days with outpatient visits, mean ± SD [median]**	**12.7 ± 16.5 [7.0]**	**8.3 ± 12.4 [4.0]**	**0.30** ^†^	
0, *n* (%)	26,487 (7.9%)	140,584 (13.9%)	0.20^†^	
1–4, *n* (%)	95,820 (28.4%)	372,909 (36.9%)	0.18^†^	
5–9, *n* (%)	75,862 (22.5%)	226,296 (22.4%)	0.00	
≥ 10, *n* (%)	138,865 (41.2%)	271,313 (26.8%)	0.31^†^	
** Number of specialist visits (i.e., psychiatrist, neurologist), mean ± SD [median]**	**1.0 ± 4.0 [0.0]**	**0.2 ± 1.8 [0.0]**	**0.24** ^†^	
0, *n* (%)	276,641 (82.1%)	954,145 (94.4%)	0.39^†^	
1–4, *n* (%)	38,941 (11.6%)	44,160 (4.4%)	0.27^†^	
5–9, *n* (%)	13,703 (4.1%)	8,502 (0.8%)	0.21^†^	
≥ 10, *n* (%)	7,749 (2.3%)	4,295 (0.4%)	0.16^†^	

ADHD, attention-deficit/hyperactivity disorder; SD, standard deviation; std. diff., standardized difference

^†^Indicates std. diff > 0.10

Notes:

^1^ Characteristics with a std. diff. >0.10 were considered as potential risk factors

^2^ Assessed during the 12 months prior to the index date

^3^ Based on Quan, H. et al., 2005. Coding Algorithms and for Defining Comorbidities Data in ICD-9-CM and ICD-10 Administrative Data. Medical Care, 43(11), pp.1130–1139; Quan, H. et al. 2011. Updating and validating the Charlson Comorbidity Index and score for risk adjustment in hospital discharge abstracts using data from 6 countries. American Journal of Epidemiology, 173(6), pp. 676–682 and Elixhauser A, Steiner C, Kruzikas. D. HCUP Comorbidity Software. Healthcare Cost and Utilization Project (HCUP). October 2015. Agency for Healthcare Research and Quality, Rockville, MD. Available from: https://www.hcup-us.ahrq.gov/toolssoftware/comorbidity/comorbidity.jsp#download

^4^ Based on American Psychiatric Pub (2022). Diagnostic and Statistical Manual of Mental Disorders (DSM-5-TR®).

^5^ The number of mental health comorbidities was calculated for the broad DSM-5 categories (i.e., multiple subtypes of a comorbidity would be counted as one). ADHD was excluded from the calculation

#### Demographic characteristics

As of index date, the ADHD cohort was younger than the non-ADHD cohort (mean age: 35.2 and 44.0 years; std. diff. = 0.68). In both cohorts, slightly over half of the patients were female (54.5% and 52.4%; std. diff. = 0.04), and the South was the most represented region (48.7% and 42.5%; std. diff. = 0.13).

#### Clinical characteristics

During the baseline period, the most frequent physical comorbidities in the ADHD and non-ADHD cohorts were hypertension (12.4% and 21.3%; std. diff. = 0.24), obesity (10.0% and 9.4%; std. diff. = 0.02), and chronic pulmonary disease (9.0% and 7.2%; std. diff. = 0.07).

A lower proportion of patients had no mental health comorbidities in the ADHD cohort than the non-ADHD cohort (42.0% and 70.8%; std. diff. = 0.61). The mean ± SD number of mental health comorbidities was 1.2 ± 1.4 in the ADHD cohort and 0.5 ± 0.9 in the non-ADHD cohort (std. diff. = 0.65). The most frequent mental health comorbidities in the ADHD and non-ADHD cohorts were anxiety disorders (34.4% and 11.1%; std. diff. = 0.58), depressive disorders (27.9% and 7.8%; std. diff. = 0.54), sleep-wake disorders (13.2% and 7.7%; std. diff. = 0.18), trauma- and stressor-related disorders (12.4% and 3.4%; std. diff. = 0.34), and substance-related and addictive disorders (9.4% and 5.0%; std. diff. = 0.17).

#### Pharmacological treatments

A higher proportion of patients in the ADHD than the non-ADHD cohort received antidepressants (40.9% and 15.8%; std. diff. = 0.58), antianxiety agents (20.6% and 8.3%; std. diff. = 0.36), anticonvulsants (16.1% and 6.8%; std. diff. = 0.29), and antipsychotics (7.2% and 1.5%; std. diff. = 0.28).

#### Healthcare resource utilization

The ADHD cohort, relative to the non-ADHD cohort, had generally higher mean ± SD rates of healthcare resource utilization, including more psychotherapy visits (2.9 ± 8.8 and 0.6 ± 4.0; std. diff. = 0.34), emergency room visits (0.6 ± 1.7 and 0.4 ± 1.2; std. diff. = 0.14), outpatient visits (12.7 ± 16.5 and 8.3 ± 12.4; std. diff. = 0.30), and specialist visits (1.0 ± 4.0 and 0.2 ± 1.8; std. diff. = 0.24); the number of inpatient visits were similar between cohorts (0.1 ± 0.4 and 0.1 ± 0.3; std. diff. = 0.04).

### Association between risk factors and ADHD diagnosis

**Fig. 2:**
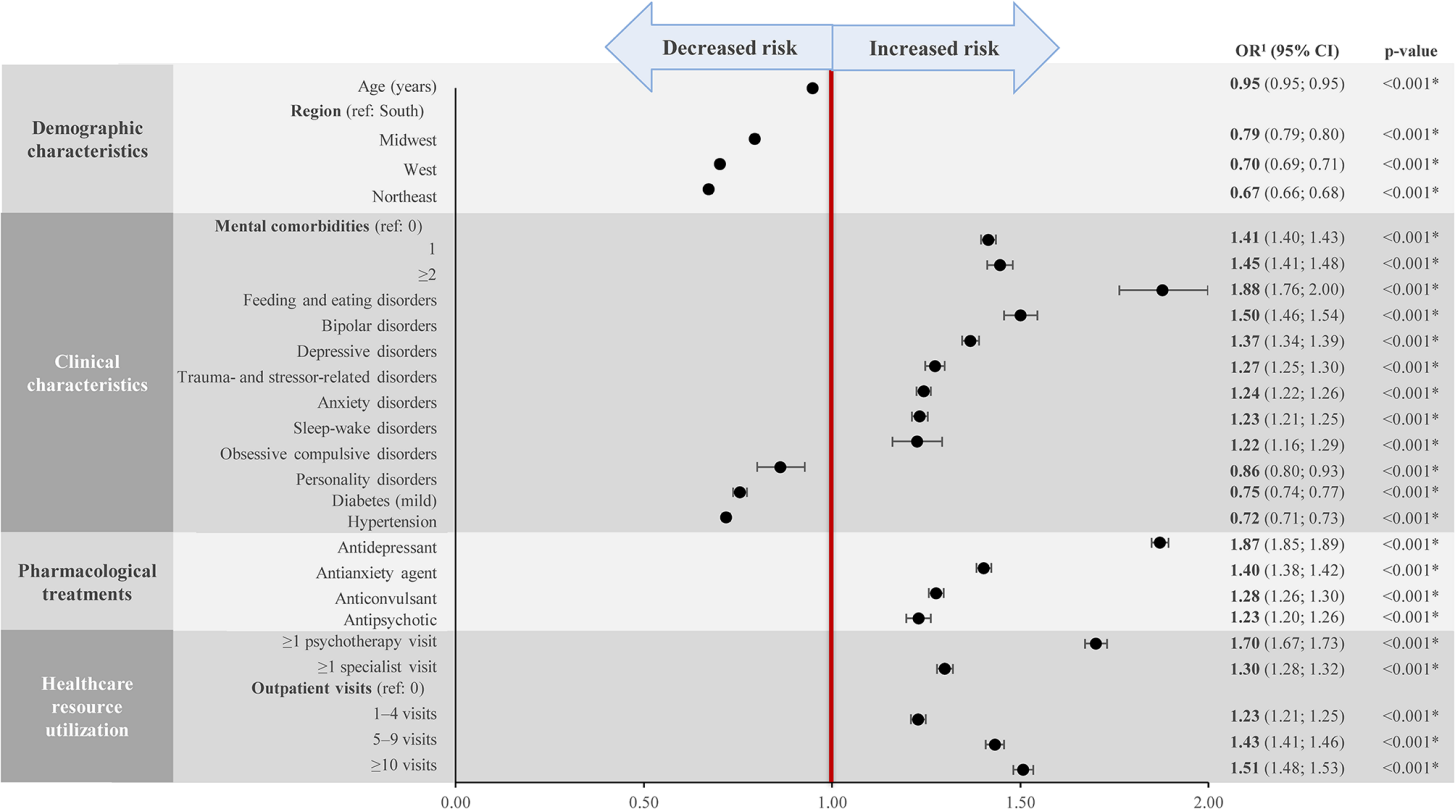
Association between risk factors and ADHD diagnosis ADHD, attention-deficit/hyperactivity disorder; CI, confidence interval; OR, odds ratio *Statistically significant at the 5% level Notes: ^1^ Estimated from logistic regression analyses

The risk factors with a significant association with an ADHD diagnosis are presented in Fig. 2. Demographically, being younger and living in the South were risk factors for having an ADHD diagnosis (OR for age: 0.95; OR for region of residence using South as a reference: Midwest, 0.79; West, 0.70; Northwest, 0.67; all p < 0.05).

Other key risk factors associated with a significantly increased probability of having an ADHD diagnosis included the number of mental health comorbidities (OR for 1 comorbidity: 1.41; ≥2 comorbidities: 1.45); certain mental health comorbidities, including feeding and eating disorders (OR: 1.88), bipolar disorders (OR: 1.50), depressive disorders (OR: 1.37), trauma- and stressor-related disorders (OR: 1.27), anxiety disorders (OR: 1.24), sleep-wake disorders (OR: 1.23), and obsessive compulsive disorders (OR: 1.22); use of antidepressants (OR: 1.87) and antianxiety agents (OR: 1.40); and having ≥ 1 psychotherapy visit (OR: 1.70), ≥ 1 specialist visit (OR: 1.30), and ≥ 10 outpatient visits (OR: 1.51) (all p < 0.05).

### Predicted risk of ADHD for patient profiles with selected risk factors

**Fig. 3:**
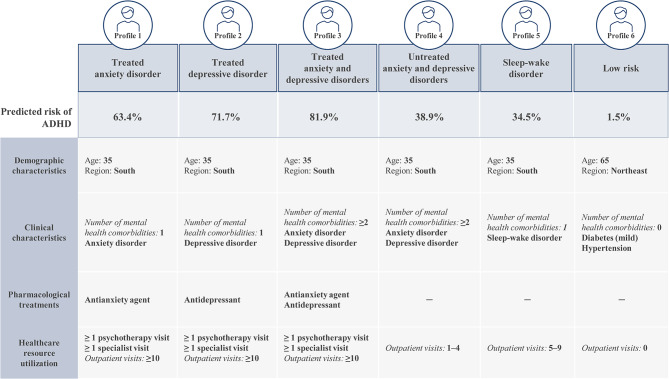
Predicted risk of ADHD for selected patient profiles ADHD, attention-deficit/hyperactivity disorder

Selected risk factors identified from the logistic regression analyses were used to create fictitious common patient profiles to demonstrate their combined impact on the predicted risk of having an ADHD diagnosis (Fig. 3). Five of the six profiles correspond to patients with the same demographic characteristics (i.e., aged 35 years and living in the South) but vary in terms of the number (i.e., 1 or ≥ 2) and types of mental health comorbidities (i.e., anxiety disorder and/or depressive disorder), the pharmacological treatment received (i.e., antianxiety and/or antidepressant agent, or no treatment), and the level of healthcare resource utilization (i.e., number of psychotherapy, specialist, and outpatient visits). The remaining profile corresponds to low-risk patients with no relevant risk factors for ADHD.

Based on these patient profiles, the predicted risk of ADHD was the highest among patients with treated anxiety and depressive disorders (profile 3). More specifically, a patient presenting with the characteristics described in this profile would have an 81.9% likelihood of being diagnosed with ADHD in the coming year. The profile with the next highest predicted risk of ADHD was patients with treated depressive disorder (profile 2; 71.7%), followed by patients with treated anxiety disorder (profile 1; 63.4%). Profiles corresponding to a moderate predicted risk of ADHD included patients with untreated anxiety and depressive disorders (profile 4; 38.9%) and patients with sleep-wake disorder (profile 5; 34.5%). The predicted risk for ADHD among low-risk patients (profile 6) was 1.5%.

## Discussion

This large retrospective case-control study has identified a broad range of risk factors associated with ADHD in adults and quantified the added likelihood of an ADHD diagnosis contributed by each factor. Certain mental health comorbidities and their associated treatments and care were found to be significantly associated with newly diagnosed ADHD in adults. Specifically, the presence of common mental health comorbidities of ADHD such as anxiety and depressive disorders was associated with 24% and 37% increased risk of having an ADHD diagnosis, respectively. The use of pharmacological treatments for these conditions such as antianxiety agents and antidepressants was associated with an increased risk of having an ADHD diagnosis of 40% and 87%, respectively; having at least one prior psychotherapy visit was also associated with a 70% increased risk. Demographically, being younger and living in the South were found to be risk factors for having an ADHD diagnosis. The combined impact of selected risk factors on the predicted ADHD risk was explored through specific patient profiles, which demonstrated how the findings may be interpreted in clinical settings. The presence of a combination of risk factors may suggest that a patient is at a high risk of having undiagnosed ADHD and signify the need for further assessments. Collectively, findings of this study have extended our understanding on the patient path to ADHD diagnosis as well as the characteristics and clinical events that could suggest undiagnosed ADHD in adults.

Most prior studies examining characteristics associated with ADHD have focused on a single or a few factors, and many were conducted in pediatric populations [18–23]. Nonetheless, the risk factors for ADHD identified in the current study are largely aligned with the literature. For instance, among prior research in adults, a multicenter patient register study found that at the time of first ADHD diagnosis, mental health comorbidities were present in two-thirds of the patients; patients on average presented with 2.4 comorbidities, with the most common comorbidities being substance use disorders, anxiety disorders, mood disorders, and personality disorders [6]. Another study among adult members of two large managed healthcare plans found that compared with individuals without ADHD, those screened positive for ADHD through a telephone survey but had no documented ADHD diagnosis (i.e., the undiagnosed group) had significantly higher rates of mental health comorbidities (e.g., anxiety, depression, bipolar disorder) and were more likely to receive medications for a mental health condition [25]. In line with these findings, the current exploratory patient profile analyses also suggest that patients with more mental health comorbidities and have received the associated pharmacological treatments and care are at a higher risk of having undiagnosed ADHD than those with fewer or untreated mental health comorbidities.

The current study also found that an overall higher healthcare resource utilization was a characteristic associated with newly diagnosed ADHD among adult patients. A potential interpretation of this finding is that an individual who experienced ADHD-related symptoms might visit a psychologist or physician frequently to seek help for the symptoms; thus, a high level of prior healthcare resource utilization may be a sign that an individual could have undiagnosed ADHD. Clinical judgement should be applied to determine whether further evaluation for ADHD is needed on a case-by-case basis considering the presence of other high-risk characteristics.

The diagnosis of ADHD can be challenging, particularly among adults [2, 3]. The current study suggests that information on patient characteristics, such as the presence of mental health comorbidities and healthcare resource utilization history, may be used to aid clinicians identify adult patients at risk of ADHD and minimize missed opportunity to provide a timely diagnosis of ADHD and the proper care. Notably, underdiagnosis or a delayed diagnosis of ADHD leads to undertreatment and can adversely affect patients’ occupational achievements, diminish self-esteem, and hamper interpersonal relationships, considerably reducing the quality of life [8]. ADHD in adults has also been shown to be associated with approximately $123 billion total societal excess costs in the US [26]. Consequently, early detection and treatment of ADHD may have the potential to alleviate the large patient and societal burden associated with the condition.

It is worth mentioning that causes for ADHD is multifactorial, and multiple risk factors may contribute to the risk of having ADHD [15]. Some risk factors in the literature (e.g., genetics and environmental factors [27, 28]) are not available in claims data, and these factors are important to consider when establishing an ADHD diagnosis. Nonetheless, the risk factors identified in this study were generated based on a large sample size (over 1.3 million adults), and as exemplified by the exploratory patient profiles, the presence of multiple risk factors was associated with an overall higher risk of having undiagnosed ADHD. Together, these findings would help inform clinicians on the types of high-risk patient profiles that should raise a red flag for potential ADHD and prompt further clinical assessments, such as family psychiatric history and diagnostic interviews. As such, findings of this study may facilitate early diagnosis and appropriate management of ADHD among adults, which may in turn improve patient outcomes.

The findings of the current study should be considered in light of certain limitations inherent to retrospective databases using claims data, including the risk of data omissions, coding errors, and the presence of rule-out diagnosis. Nonetheless, while few studies specifically assessed the validity of ICD-10-CM codes for ADHD diagnoses in claims data, literature evidence has suggested high accuracy of ICD-9-CM codes in identifying neurodevelopmental disorders, including ADHD, and a good correspondence between the ICD-9 and − 10 codes is expected [29, 30]. Furthermore, ICD codes have been widely used in the literature to identify ADHD diagnoses in claims-based analyses [31–33]. Meanwhile, as the study included commercially insured patients, the sample may not be representative of the entire ADHD population in the US. Furthermore, potential risk factors were limited to information available in health insurance claims data only, which may lack relevant information related to ADHD, such as presence of childhood ADHD, family history, or environmental factors. In addition, some characteristics may interact with multiple variables such that their association with an ADHD diagnosis may already be captured by other variables; as such, a characteristic with an OR of less than 1 should not be interpreted as having a protective effect against an ADHD diagnosis but rather that the characteristic alone may be insufficient to prompt screening for ADHD. Lastly, findings from this retrospective observational analysis should be interpreted as measures of association; no causal inference can be drawn.

## Conclusions

This large retrospective case-control study found that mental health comorbidities and related treatments and care are significantly associated with newly diagnosed ADHD in US adults. The presence of a combination of risk factors may suggest that a patient is at a high risk of having undiagnosed ADHD. The results of this study provide insights on the path to ADHD diagnosis and may aid clinicians identify at-risk patients for screening, which may facilitate early diagnosis and appropriate management of ADHD.

## Data Availability

The data that support the findings of this study are available from IQVIA but restrictions apply to the availability of these data, which were used under license for the current study, and so are not publicly available. Data are however available from the corresponding author (email: Rebecca.Bungay@analysisgroup.com) upon reasonable request and with permission of IQVIA.

## Declarations

### Previous presentation

Part of the material in this manuscript was presented at the International Society for Pharmacoeconomics and Outcomes Research (ISPOR) 2023 conference held on May 7–10, 2023, in Boston, MA, as a poster presentation.

## List of abbreviations

ADHD: Attention-deficit/hyperactivity disorder
CI: Confidence intervals
HIPAA: Health Insurance Portability and Accountability Act
ICD-10-CM: International Classification of Diseases, Tenth Revision, Clinical Modification
OR: Odds ratio
SD: Standard deviation
Std. diff: Standardized difference
US: United States
